# Intracranial dural arteriovenous fistulas with and without pial arterial supply: a case series of 227 patients

**DOI:** 10.3389/fneur.2026.1872358

**Published:** 2026-08-17

**Authors:** Shenyun Yuan, Xinjun Chen, Wenyuan Zhao, Yu Feng, Yanyan Wu, Hao Chen, Wei Wei, Yihui Ma

**Affiliations:** Department of Neurosurgery, Zhongnan Hospital of Wuhan University, Wuhan, China

**Keywords:** DAVF, endovascular treatment, hybrid surgery, microsurgery, pial arterial supply

## Abstract

**Background:**

Intracranial dural arteriovenous fistulas (DAVFs) with pial arterial supply (PAS) constitute a distinct subgroup characterized by more complex angioarchitecture and potential implications for treatment planning. This study evaluated the incidence, angiographic characteristics, and clinical predictors of PAS in a cohort of patients with DAVFs.

**Methods:**

This retrospective study included 227 consecutive patients with intracranial DAVF treated at our institution. Clinical and imaging data were reviewed and analyzed to characterize DAVF angioarchitecture and determine the presence of PAS.

**Results:**

A total of 227 patients with DAVFs were included, and PAS was identified in 44 patients (19.4%). DAVFs with PAS were more frequently located at the cerebral convexity and tentorium than DAVFs without PAS. In addition, DAVFs with PAS were associated with higher angiographic grades and greater venous dilation. Clinical presentation rates were comparable between the two groups. Fistulas with PAS were more often managed with endovascular treatment. Microsurgery and hybrid surgery may represent effective treatment options in selected patients with complex DAVFs such as DAVFs located in the anterior cranial fossa (ACF) or foramen magnum (FM). Rates of surgical disconnection or hybrid surgery were similar between the two groups. Compared with DAVFs without PAS, DAVFs with PAS had significantly lower complete obliteration rates. Major complication rates, including postoperative ICH and cerebral infarction, were similar between the groups.

**Conclusion:**

DAVFs with PAS are associated with higher angiographic grades and increased venous dilation. DAVFs with PAS pose unique therapeutic challenges, as reflected by lower complete obliteration rates. Nevertheless, with careful technique and appropriate surgical strategies, major complication rates can be comparable to those of DAVFs without PAS.

## Introduction

Intracranial dural arteriovenous fistulas (DAVFs) are abnormal vascular shunts connecting a dural artery to a dural venous sinus and/or leptomeningeal veins (1). DAVFs are rare neurovascular lesions that account for 10–15% of all intracranial vascular malformations and have a broad clinical spectrum, ranging from benign symptoms to aggressive neurological complications, such as intracranial hemorrhage (ICH) or progressive neurological deficits (2). The highly variable angioarchitecture of DAVFs plays a critical role in determining their natural history and guiding therapeutic strategies (3). The main classifications of DAVFs are the Borden and Cognard systems (4). Both are based on venous drainage patterns, specifically the presence of cortical venous reflux (CVR), which determines the lesion’s clinical risk.

DAVFs are traditionally regarded as lesions supplied predominantly by dural branches of the external carotid artery (ECA), along with meningeal branches of the internal carotid artery (ICA) and vertebral artery (VA) (5). However, several reports have shown that pial arterial feeders may also contribute to the supply of DAVFs, particularly in complex or high-flow fistulas (6, 7). The presence of pial arterial supply (PAS) is clinically important because it may influence lesion angioarchitecture and treatment planning and may increase the risk of ischemic complications during endovascular treatment (8, 9). The mechanisms underlying PAS in DAVFs remain poorly understood. One hypothesis is that chronic venous hypertension associated with DAVFs promotes angiogenesis and recruitment of cortical arterial branches (10–12).

Previous retrospective studies evaluating prevalence, clinical factors, and predictors have yielded conflicting findings regarding the true risks associated with PAS (8, 13, 14). Therefore, this study aimed to evaluate the incidence, angiographic characteristics, treatment outcomes, and clinical predictors of PAS in a cohort of patients with DAVFs.

## Materials and methods

### Patients

Between October 2017 and October 2025, 227 consecutive patients with intracranial DAVF at our institution were retrospectively investigated. Patients with direct carotid-cavernous fistula or spinal DAVF were excluded. Clinical symptoms, demographic data, DAVF characteristics, and treatment procedures were collected and analyzed. This study was approved by the ethics committee of our institution.

### Imaging acquisition and analysis

For assessment of the DAVF angioarchitecture, two independent experienced neurosurgeons (both of whom have more than 10 years of working experience) performed this review. The diagnosis of DAVF with PAS was established based on imaging findings and clinical manifestations. PAS was classified into two categories: pre-existing dural branches, defined as dilated pre-existing dural branches of pial arteries, and pure pial supply, defined as tortuous and ramified vessels located outside known physiological connections between pial arteries and the dura. This classification system was previously used by Osada and Krings (14). Additional angioarchitectural data were collected and are presented in Table 1, including Borden classification, Cognard classification, fistula location, cortical venous dilation, and venous sinus stenosis or thrombosis. Cortical venous dilation refers to an abnormal widening (dilatation) of the veins that run on the surface of the brain.

**Table 1:** Comparison of patients with and without pial arterial supply of intracranial DAVFs.

Variable	With pial arterial supply (*n* = 44)	Without pial arterial supply (*n* = 183)	*p* value (univariate analysis)	OR (95% CI)	Multivariate analysis	
					OR (95% CI)	*p* value
Age (mean ± SD), yr	54.9 ± 10.7	56.3 ± 12.6	0.455	-		
Female sex	18 (40.91%)	78 (42.62%)	0.836	0.93 (0.48–1.82)		
Location of DAVF						
Torcular	3 (6.82%)	5 (2.73%)	0.187	2.61 (0.60–11.34)		
CS	0	40 (21.86%)	*p* < 0.001	-		
Convexity	16 (36.36%)	16 (8.74%)	*p* < 0.001	5.96 (2.68–13.28)	5.12 (2.01–13.04)	P < 0.001
TSS	5 (11.36%)	37 (20.22%)	0.174	0.51 (0.19–1.37)		
FM	1 (2.27%)	25 (13.66%)	0.035	0.15 (0.02–0.98)	0.12 (0.02–0.81)	0.030
ACF	4 (9.09%)	25 (13.66%)	0.415	0.63 (0.21–1.92)		
SSS	0	4 (2.19%)	1.000	-		
Tentorium	12 (27.27%)	26 (14.21%)	0.037	2.26 (1.04–4.95)	1.58 (0.61–4.10)	0.348
Multiple	3 (6.82%)	4 (2.19%)	0.134	3.27 (0.71–15.20)		
Others	0	1 (0.55%)	1.000	-		
Borden classification						
I	0	38 (20.77%)	*p* < 0.001	-		
II	3 (6.82%)	51 (27.87%)	0.003	0.19 (0.06–0.64)		
III	41 (93.18%)	94 (51.37%)	*p* < 0.001	12.94 (3.87–43.29)		
Cognard classification						
I + IIa	0	57 (31.15%)	*p* < 0.001	-		
IIa+b, IIb, III	5 (11.36%)	30 (16.39%)	0.407	0.65 (0.24–1.80)		
IV/V	39 (88.64%)	96 (52.46%)	*p* < 0.001	7.07 (2.67–18.74)	3.87 (1.32–11.35)	0.014
Imaging findings						
Cortical venous dilation	41 (93.18%)	120 (65.57%)	*p* < 0.001	7.18 (2.14–24.09)	2.34 (0.64–8.57)	0.201
Venous sinus stenosis/occlusion	10 (22.73%)	52 (28.42%)	0.447	0.74 (0.34–1.61)		
Presentation						
Intracranial hemorrhage	15 (34.09%)	80 (43.72%)	0.245	0.67 (0.34–1.33)		
Aggressive symptoms	25 (56.82%)	108 (59.02%)	0.790	0.91 (0.47–1.78)		

Aggressive symptoms comprised ICH and nonhemorrhagic neurological deficits. CS: Cavernous sinus; TSS: Transverse-sigmoid sinus; FM: Foramen magnum; ACF: Anterior cranial fossa; SSS: Superior sagittal sinus.

### Treatment strategy

**Figure 1:**
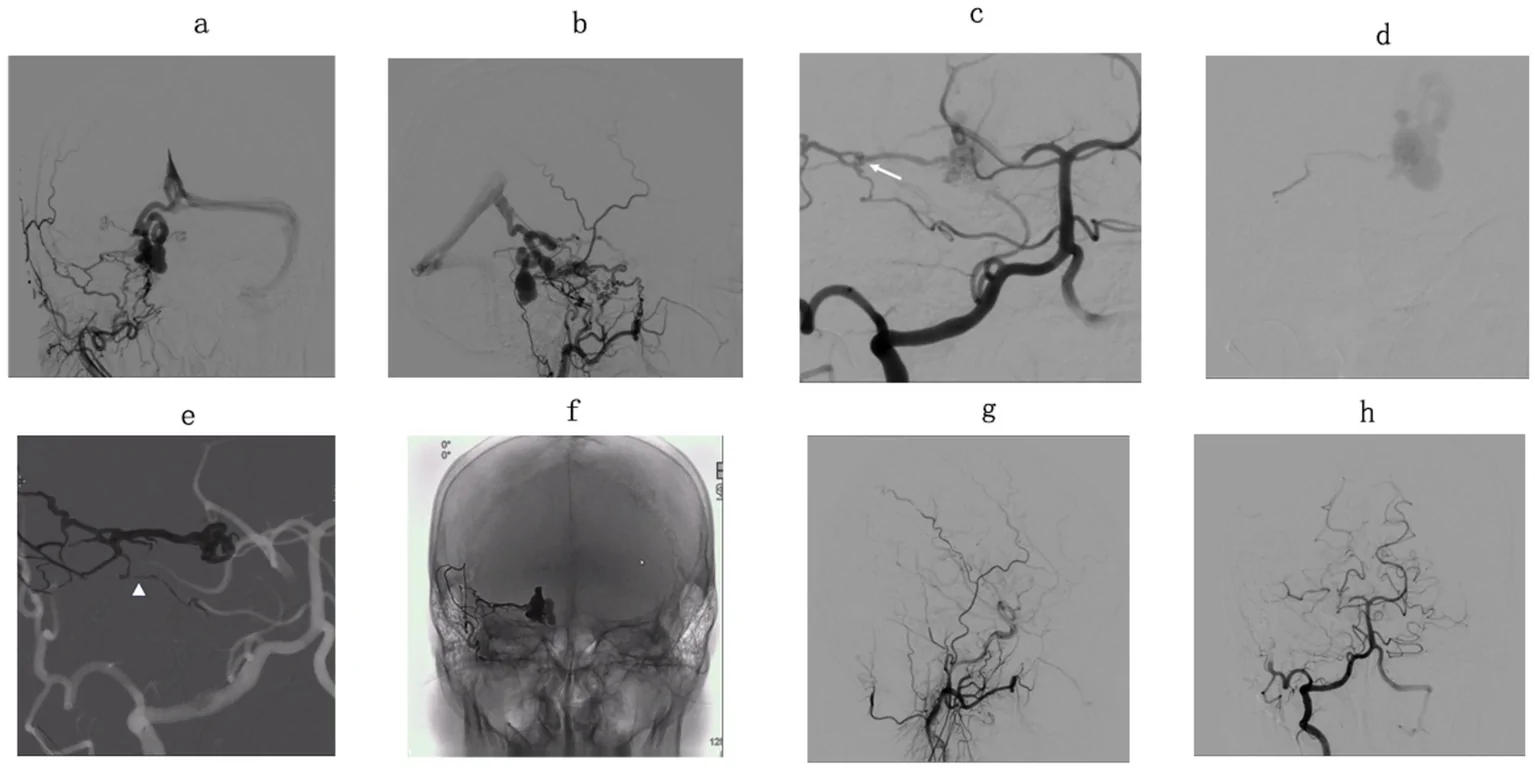
Example of endovascular treatment for tentorial DAVF. **(a,b)** Preoperative external carotid artery (ECA) arteriograms show a tentorial DAVF draining into the basilar vein. **(c)** AP view of the right vertebral artery (VA) arteriogram shows a fistula feeder, a subarcuate artery branch of the AICA (white arrow). **(d)** Selective middle meningeal artery (MMA) arteriogram shows a high-flow fistula draining into a venous pouch. **(e)** Onyx refluxed into the pial supply of the fistula (white arrowhead). **(f)** Onyx cast after treatment via the right MMA. **(g,h)** Immediate postoperative ECA arteriograms show complete obliteration of the fistula.

**Figure 2:**
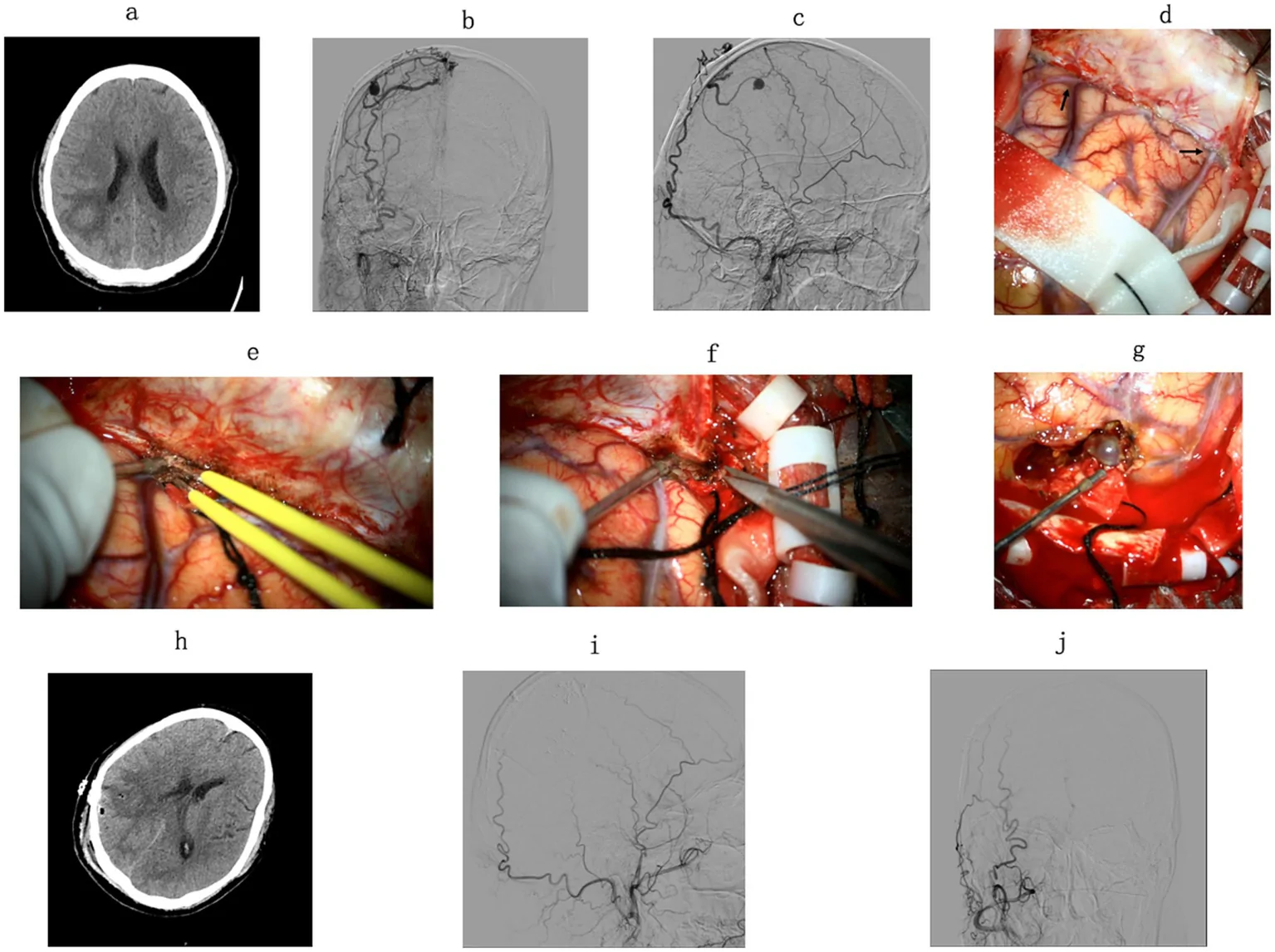
Example of microsurgical treatment for convexity DAVF. **(a)** Brain computed tomography (CT) reveals an intraparenchymal hemorrhage. **(b,c)** AP and lateral ECA arteriograms show a convexity DAVF draining into cortical veins. **(d)** Intraoperative view of the right parietal lobe shows arterialized cortical veins (black arrow). **(e–g)** Fistula disconnection is completed by coagulating the draining veins distal to the fistulous point. **(h)** Postoperative non-contrast axial CT confirms coagulation of the venous pouch. **(i,j)** Six-month follow-up angiograms demonstrate persistent obliteration of the fistula.

**Figure 3:**
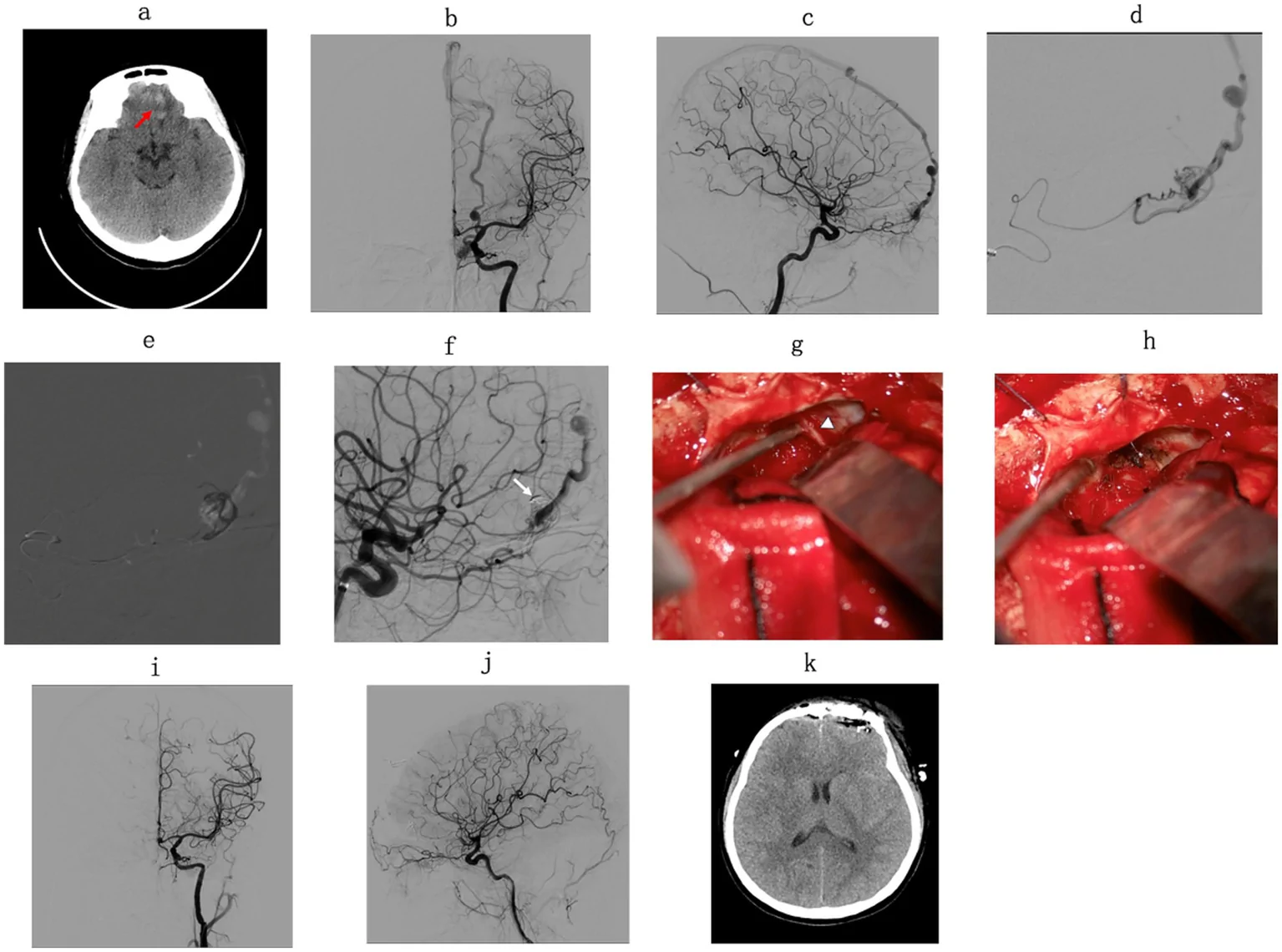
Example of hybrid surgical treatment for anterior cranial fossa (ACF) DAVF. **(a)** Brain CT reveals an intraparenchymal hemorrhage. **(b,c)** AP and lateral internal carotid artery (ICA) arteriograms show the ACF DAVF draining into a frontal vein. **(d)** Selective ophthalmic artery arteriogram shows the fistula draining into a venous pouch. **(e,f)** Angiograms reveal the Onyx cast and partial embolization of the fistula. **(g,h)** Intraoperative views of the ACF show the arterialized vein and disconnection of the fistula by coagulation of the draining vein distal to the fistulous point. **(i,j)** Immediate angiograms demonstrate complete obliteration of the fistula. **(k)** Postoperative non-contrast axial CT shows embolic material close to the frontal lobe.

Therapeutic risks and the probability of cure were evaluated through discussion by our institutional cerebrovascular board. In general, endovascular treatment (Figure 1), including transarterial embolization and transvenous embolization, was considered the first-line option, whereas microsurgery was considered the second-line option (Figure 2). When no appropriate route was available, and curative embolization could not be achieved, one-stop hybrid surgery combining preoperative embolization with microsurgical obliteration (Figure 3) was considered. Both intraoperative angiography and endovascular embolization were performed using a biplane flat-panel angiographic suite (Allura Xper FD20; Philips, Amsterdam, The Netherlands).

During endovascular embolization, embolization was performed through the microcatheter using n-butyl cyanoacrylate (TruFill n-butyl cyanoacrylate (n-BCA); Cordis Neurovascular, Miami Lakes, Florida, United States), ethylene vinyl copolymer (Onyx 18; Medtronic, Minneapolis, Minnesota, United States), and detachable coils. During microsurgery, surgical approaches were selected according to the location of the cortical venous drainage (CVD). After adequate exposure of the target CVD, we generally attempted CVD disconnection with a surgical clip or coagulation at the fistulous point of the draining veins from the dura. During one-stop hybrid microsurgery, intraoperative digital subtraction angiography (DSA) was performed during surgery and before craniotomy closure to confirm total obliteration of the fistula. Patients who underwent treatment were analyzed for occlusion rate. Patients underwent DSA before embolization, immediately after treatment, and 3–6 months follow-up after treatment. The patient’s improvement in clinical symptoms was followed up one year after treatment. Angiographic outcomes were defined as complete occlusion when no residual fistula was present and partial occlusion when a residual fistula persisted as shown in the immediate postoperative angiography.

### Statistical analysis

Results are reported as means with standard deviations or as percentages. The Student’s t-test was used to analyze continuous variables, whereas the Chi-square test was used to analyze categorical variables. We used variables, in which *p* values were less than 0.05 in the univariate analyses, to perform the multivariate analysis. Fisher’s exact test was used to compare the frequencies of perioperative complications and presentation of patient with PAS. For all statistical analyses, *p* < 0.05 was considered statistically significant.

## Results

### Patient and fistula characteristics

A total of 227 patients with intracranial DAVFs were included in this study (Table 1). PAS was identified in 44 patients (19.4%), and was absent in 183 patients (80.6%). The mean age of patients with PAS (54.9 ± 10.7 years) was lower than that of patients without PAS (56.3 ± 12.6 years), although the difference was not statistically significant (*p* = 0.45). Gender distribution also did not differ significantly between the two groups. Fistulas without PAS were located in the transverse-sigmoid sinus (TSS) in 37 patients, cavernous sinus (CS) in 40, tentorium in 26, convexity in 16, foramen magnum (FM) in 25, anterior cranial fossa (ACF) in 25, superior sagittal sinus (SSS) in 4, and multiple sites in 4. Fistulas with PAS were located in the TSS in 5 patients, CS in 0, tentorium in 12, convexity in 16, torcular region in 3, FM in 1, and multiple sites in 3. Compared with DAVFs without PAS, DAVFs with PAS were more likely to be located at the cerebral convexity (36.36% vs. 8.7%, *p* < 0.001). Tentorial DAVFs were observed in 12 patients (27.27%) in the PAS group and in 26 patients (14.21%) in the non-PAS group (*p* < 0.05). FM DAVFs were observed in 26 patients (14.1%) in the non-PAS group and in 1 patient (2.3%) in the PAS group (*p* < 0.05). Compared with DAVFs without PAS, DAVFs with PAS were more likely to present as high-grade Borden III lesions (93.18% vs. 51.37%, *p* < 0.001) and high-grade Cognard IV/V lesions (88.64% vs. 52.46%, *p* < 0.001). CVD was observed in 41 patients with PAS (93.18%) and in 120 patients without PAS (65.57%; *p* < 0.001). Regarding clinical presentation, the incidence of ICH and other aggressive symptoms did not differ significantly between the two groups. The multivariate analysis identified 3 independent predictors of a PAS: convexity (*p* < 0.001; OR: 5.12), FM (*p* < 0.05; OR: 0.12), and high-grade Cognard IV/V lesions (*p* < 0.05; OR: 3.87).

Pial arterial supplies were classified into 2 categories based on morphological and anatomic findings including pure pial supply and dilated durtical branches (Table 2). Among the 44 patients with PAS, 19 had one or more dilated dural branches of the pial artery, and 25 had pure pial supply. Among these patients, 43 patients underwent treatment, and 2 patients had both dilated dural branches and pure pial supply (Table 2). The mean age of patients with pure pial supply was 53.8 ± 12.9 years, which was lower than that of patients with a dilated dural branch of the pial artery (55.9 ± 11.1) years; however, this difference was not significant. Among patients with a dilated pre-existing dural branch of a pial artery, 8 cases (42.1%) were located in the tentorium, whereas 4 patients (16%) with pure pial supply had tentorial fistulas. Conversely, 4 of 24 patients with pure pial supply (16%) had ACF DAVFs, whereas no ACF fistula was observed among patients with a dilated pre-existing dural branch of a pial artery.

**Table 2:** Characteristics of DAVFs with pial arterial supply within each classification.

Variable	Classification of pial arterial supply		*p* value (univariate analysis)	OR (95% CI)
	Dilated Dural branch (*n* = 19)	Pure pial supply (*n* = 25)		
Age (mean ± SD), yr	55.9 ± 11.1	53.8 ± 12.9	0.566	-
Supplying arteries	MMA (*n* = 15)	MMA (*n* = 20)		
	AICA (*n* = 5)	MCA (*n* = 11)		
	OphA (*n* = 5)	ACA (*n* = 6)		
	MHT (*n* = 4)	PCA (*n* = 5)		
	PCA (*n* = 4)	OphA (*n* = 5)		
	PMA (*n* = 4)	OA (*n* = 4)		
	PICA (*n* = 3)	AICA (*n* = 3)		
	SCA (*n* = 2)	MHT (*n* = 3)		
	MCA (*n* = 0)	SCA (*n* = 3)		
Morphological feature	Linear vessel	Tortuous and ramified vessel		
Intracranial hemorrhage	8 (42.11%)	11 (44.00%)	1.000	0.93 (0.26–3.24)
Aggressive symptoms	10 (52.63%)	15 (60.00%)	0.759	0.74 (0.22–2.47)
Location of DAVF	Torcular (*n* = 3)	Torcular (*n* = 0)		
	Convexity (*n* = 4)	Convexity (*n* = 12)		
	TSS (*n* = 4)	TSS (*n* = 1)		
	ACF (*n* = 0)	ACF (*n* = 4)		
	Tentorium (*n* = 8)	Tentorium (*n* = 4)		
	Multiple (*n* = 0)	Multiple (*n* = 3)		
	FM (*n* = 0)	FM (*n* = 1)		

TSS: transverse-sigmoid sinus; ACF: anterior cranial fossa; FM: foramen magnum; MMA: middle meningeal artery; AICA: anterior inferior cerebellar artery; MHT: meningohypophyseal trunk; PCA: posterior cerebral artery; PICA: posterior inferior cerebellar artery; SCA: superior cerebellar artery; MCA: middle cerebral artery; ACA: anterior cerebral artery; OA: occipital artery; PMA: posterior meningeal artery; OphA: ophthalmic artery.

### Treatment results

Of the 227 patients, 212 underwent treatment (93.4%), and 15 were managed conservatively (6.6%, Table 3). Embolization alone, surgery alone, hybrid surgery, and conservative management were performed in 147 (64.8%), 43 (18.9%), 22 (9.7%), and 15 (6.6%) patients, respectively. Fistulas with PAS were more likely to be treated with endovascular therapy (77.27% vs. 61.75%, *p* = 0.053), although this difference was not significant. Rates of surgical disconnection or hybrid surgery were similar between the pial and non-pial supply groups. Among these patients, 212 patients underwent treatment and obliteration rates were calculated (Table 3). One hundred and seventy-six patients came to our institution for clinical follow-up, of whom one hundred and twenty-one had complete imaging data including cerebral angiography. Compared with DAVFs without PAS, DAVFs with PAS (76.74% vs. 95.27%, *p* < 0.001) and high-grade Cognard or Borden lesions showed lower complete obliteration rates (Tables 3, 4). The multivariate analysis also confirmed that DAVFs with PAS, high-grade Cognard lesions (IIa + b, IIb, III) and venous sinus stenosis/occlusion had lower complete obliteration rates (Table 4). One year later, all patients with followed up DSA showed no recurrence. Major complication rates, including postoperative ICH and cerebral infarction, were similar between the PAS and non-PAS groups. Among patients with ICH, two PAS cases experienced intraoperative perforation, but neither resulted in a permanent deficit. Four patients experienced minor postoperative cerebral infarction, which did not result in neurological dysfunction.

**Table 3:** Treatment characteristics and outcomes in patients with and without pial arterial supply of intracranial DAVFs.

Variable	With pial arterial supply (*n* = 44)	Without pial arterial supply (*n* = 183)	*p* value univariate analysis	OR (95% CI)
Treatment modality				
Conservative treatment	1 (2.27%)	14 (7.65%)	0.206	0.28 (0.01–1.92)
Embolization alone	34 (77.27%)	113 (61.75%)	0.053	2.11 (0.98–4.53)
Surgery alone	6 (13.64%)	37 (20.22%)	0.306	0.61 (0.25–1.59)
Hybrid surgery	3 (6.82%)	19 (10.38%)	0.582	0.63 (0.18–2.24)
Immediate angiographic outcomes				
Obliteration	33 (76.74%)	161 (95.27%)	*p* < 0.001	0.16 (0.07–0.37)
Reduction	10 (23.26%)	8 (4.73%)	*p* < 0.001	6.08 (2.28–16.19)
Perioperative complications				
Intraoperative hemorrhage	2 (4.55%)	0	0.036	-
Postoperative hemorrhage	1 (2.27%)	1 (0.55%)	0.351	-
Cerebral infarction	2 (4.55%)	2 (1.09%)	0.170	-
Follow-up angiographic outcomes				
Obliteration	16 (76.19%)	94 (94.00%)		
Reduction	5 (23.81%)	6 (6.00%)		
Recurrence	0	0	1.000	-
Follow-up clinical outcomes				
Complete resolution	18 (58.06%)	78 (53.79%)	0.665	1.19 (0.54–2.61)
Improvement	9 (29.03%)	55 (37.93%)	0.350	0.67 (0.29–1.56)
Deterioration	4 (12.90%)	12 (8.28%)	0.487	1.64 (0.49–5.48)

**Table 4:** Predictive factors associated with complete obliteration.

Variable	Complete obliteration (*n* = 194)	Incomplete obliteration (*n* = 18)	*p* value univariate analysis	OR (95% CI)	Multivariate analysis	
					OR (95% CI)	*p* value
Age≥70	16 (8.25%)	4 (22.22%)	0.129	0.62 (0.94–10.80)		
Female sex	63 (32.47%)	8 (44.44%)	0.306	1.66 (0.63–4.42)		
Location of DAVF						
Torcular	8 (4.12%)	0	0.630	-		
CS	37 (19.07%)	0	0.066	-		
Convexity	29 (14.95%)	2 (11.11%)	0.930	0.71 (0.16–3.26)		
TSS	30 (15.46%)	8 (44.44%)	0.006	4.37 (1.60–12.0)	2.41 (0.76–7.66)	0.136
FM	24 (12.37%)	0	0.220	-		
ACF	28 (14.43%)	1 (5.56%)	0.490	0.35 (0.04–2.73)		
SSS	3 (1.55%)	0	0.770	-		
Tentorium	30 (15.46%)	5 (27.78%)	0.310	2.10 (0.70–6.33)		
Multiple	4 (2.06%)	2 (11.11%)	0.081	5.94 (0.68–38.2)		
Others	1 (0.52%)	0	0.920	-		
Borden classification						
I	30 (15.46%)	3 (16.67%)	0.840	1.09 (0.30–4.01)		
II	42 (21.65%)	8 (44.44%)	0.059	2.90 (1.08–7.80)		
III	122 (62.89%)	7 (38.89%)	0.057	0.38 (0.13–1.05)		
Cognard classification						
I + IIa	49 (25.26%)	2 (11.11%)	0.290	0.37 (0.08–1.67)		
IIa+b, IIb, III	23 (11.86%)	9 (50.00%)	P < 0.001	7.44 (2.68–20.6)	5.89 (1.92–18.1)	0.002
IV/V	124 (63.92%)	7 (38.89%)	0.037	0.36 (0.13–0.97)	0.48 (0.17–1.36)	0.166
Imaging findings						
Cortical venous dilation	137 (70.62%)	14 (77.78%)	0.522	1.46 (0.46–4.61)		
Venous sinus stenosis/occlusion	46 (23.71%)	11 (61.11%)	0.002	5.06 (1.85–13.79)	4.16 (1.36–12.7)	0.012
PAS	33 (17.01%)	10 (55.56%)	P < 0.001	6.10 (2.24–16.62)	5.38 (1.74–16.6)	0.003

CS: Cavernous sinus; TSS: Transverse-sigmoid sinus; FM: Foramen magnum; ACF: Anterior cranial fossa; SSS: Superior sagittal sinus; DAVF: Anterior cranial fossa; PAS: pial arterial supply.

## Discussion

DAVFs with PAS represent a distinct subgroup characterized by more complex angioarchitecture and a different anatomical distribution. In this study, PAS was observed in 19.4% of patients with intracranial DAVFs. Osada et al. reported a rate of 11.3% in a series of 204 patients with intracranial DAVFs (14). Su et al. reported a 23.5% rate in a retrospective study of intracranial DAVFs (15).

This study showed that patients with PAS had a lower mean age than those without PAS, although the difference did not reach statistical significance. This trend toward younger age in the PAS group is consistent with the findings of Osada and Krings, who identified younger age as an independent predictor of pial arterial supply in multivariate analysis (14). The pathophysiology underlying this age association remains unclear but may be related to greater hemodynamic plasticity or differences in angiogenic capacity in younger patients. Furthermore, gender distribution did not differ significantly between the two groups, suggesting that pial recruitment occurs independently of sex-related factors.

Regarding location, tentorial and convexity DAVFs were more commonly observed among lesions with PAS. In contrast, fistulas without PAS were more commonly located in the CS and FM. The reason for this difference in anatomical distribution remains unclear, but it may reflect underlying anatomical or hemodynamic differences (16–18). A rich physiological anastomotic network may exist between dural and pial arterial systems along the cerebral convexity (19). Under pathological conditions involving venous hypertension or sustained dural arteriovenous shunting, these pre-existing anastomoses may dilate and recruit pial arterial flow to supplement fistula perfusion (20, 21).

A key observation in this study was that DAVFs with PAS were associated with higher angiographic grades and greater venous dilation. DAVFs with PAS were more frequently classified as Borden type III and Cognard types IV/V, both of which are characterized by CVD and a higher risk of aggressive clinical behavior. CVD is recognized as one of the most important risk factors for hemorrhage and neurological deficits (22). Consistent with this observation, CVD was significantly more common in the PAS group than in the non-PAS group. The relationship between PAS and chronic venous hypertension (CVH) represents a plausible biological mechanism in the pathophysiology of DAVFs. The progression from CVH to the recruitment of PAS is likely to be a biological framework that explains the natural history and therapeutic challenges of aggressive DAVFs.

Despite these radiographic differences, clinical presentation rates, including ICH and other aggressive symptoms, were similar between the two groups. Compared with the non-PAS group, the PAS group had slightly lower rates of aggressive symptoms. However, Su et al. reported significant differences in clinical presentation between the two groups (15). Several factors may explain this apparent discrepancy. The sample size may have limited the ability to detect clinical differences or determine whether early diagnosis and treatment mitigated the expected increase in clinical severity in PAS cases. Alternatively, PAS may represent a radiographic marker of risk that precedes overt clinical deterioration.

In our study, DAVFs with PAS were more frequently treated using endovascular techniques. However, despite this preference, the PAS group had lower complete obliteration rates than the non-PAS group. Several factors may explain this finding. First, PAS often indicates a more complex, higher-grade fistula associated with more aggressive venous drainage patterns, including direct cortical venous reflux and venous ectasia (23). These features create high-flow, high-pressure conditions that may limit the penetration and durability of liquid embolic agents. Second, pial feeders may be difficult to embolize completely because of their small caliber, tortuosity, and risk of nontarget embolization into the normal cortical circulation (24). Third, the angiographic definition of complete obliteration requires rigorous post-treatment imaging, typically including delayed follow-up angiography (25). In PAS-positive lesions, small residual shunts supplied by pial collaterals may be overlooked on intraoperative or immediate postprocedural angiography and become apparent only on follow-up imaging.

Comparable rates of surgical disconnection or hybrid surgery between the two groups suggest that the decision to choose surgery depends primarily on factors other than PAS. A recent meta-analysis by Raki reported that treatment outcomes for cranial DAVFs are highly dependent on anatomical location (26). Microsurgery and hybrid surgery may represent effective treatment options in selected patients with complex DAVFs, particularly lesions with large venous ectasia requiring decompression, extensive arterial recruitment, or refractoriness after failed endovascular treatment (27, 28). In our study, hybrid surgery also achieved high one-stage obliteration rates for refractory DAVFs.

In this study, complication rates, including postoperative ICH and cerebral infarction, did not differ between fistulas with and without PAS. Previous studies have proposed embolizing pial feeders first to prevent intraprocedural hemorrhage (11, 29). However, the recent study by Su et al. argues against this approach, demonstrating that embolization of PAS before DAVF closure may significantly increase both hemorrhagic and ischemic complications and therefore does not support routine preclosure PAS embolization (15). Our findings of comparable major complication rates and absence of permanent deficits from perforations suggest that a selective rather than routine approach to PAS management may be appropriate. Due to the small sample size, it may be underpowered to detect meaningful differences in complication rates. Furthermore, even partial treatment or a residual fistula may achieve clinical stabilization if venous drainage patterns are favorably altered.

In a retrospective cohort study, Brinjikji also found that major complication rates were similar between the PAS and non-PAS groups (13). However, some studies have suggested that DAVFs with PAS are more likely to have treatment-related complications than those without pial supply (11, 15). This discrepancy may be explained by evolving treatment strategies, increased operator experience, or improved patient selection.

In this study, four patients experienced minor postoperative cerebral infarction without subsequent neurological dysfunction. Ischemic complications in DAVFs with PAS may arise through several mechanisms, including reflux of embolic material into pial arteries, retrograde thrombosis of pial arteries, and thromboembolic events. Hetts et al. specifically highlighted the risk of perioperative ischemic stroke in DAVFs with pial supply, noting that retrograde thrombosis of pial arteries can lead to cerebral ischemia (8). The absence of permanent deficits in our minor infarction cases likely reflects the limited territory affected or the presence of collateral circulation. This observation also suggests that ischemic risk can be acceptably managed with careful technique, including advancing microcatheters close to the fistula point and avoiding reflux of embolic agents.

The occurrence of intraoperative perforation exclusively in the PAS group (two cases) warrants particular attention. This finding supports the concept that pure pial feeders are intrinsically more fragile than dural feeders (30). Importantly, both perforations in our series resolved without permanent deficit, suggesting that prompt recognition and management of this complication can mitigate long-term morbidity.

### Limitations

This study has several limitations. First, the retrospective, single-center design may have introduced potential bias. Second, the small sample size may have been insufficient to capture the true incidence of complications. Finally, long-term follow-up angiography was not performed in all patients, which may have underestimated the true rate of residual or recurrent fistulas, particularly among those with PAS.

## Conclusion

In this study, DAVFs with PAS were associated with higher angiographic grades and increased venous dilation, reflecting the complex angioarchitecture of these lesions. DAVFs with PAS pose unique therapeutic challenges, as reflected by lower complete obliteration rates. However, with careful technique and appropriate surgical strategies, major complication rates can be comparable to those of DAVFs without PAS. Prospective multicenter studies are needed to assess the angioarchitecture of subgroup of DAVFs and determine the associations between the optimized management strategy and the clinical presentations and outcomes.

## Data Availability

The original contributions presented in the study are included in the article/supplementary material, further inquiries can be directed to the corresponding author.
